# TRIM3 and TRIM16 as potential tumor suppressors in breast cancer patients

**DOI:** 10.1186/s13104-022-06193-y

**Published:** 2022-09-30

**Authors:** Mohammad Reza Roshanazadeh, Maryam Adelipour, Arash Sanaei, Hadi Chenane, Mojtaba Rashidi

**Affiliations:** 1 Cancer Research Center, Ahvaz Jundishapur University of Medical Sciences, Ahvaz, Iran; 2 Department of clinical biochemistry, Faculty of medicine, jundishapour University of medical sciences, Ahvaz, Iran

**Keywords:** *TRIM3*, *TRIM16*, Breast cancer, Tumor suppressor, Invasion

## Abstract

**Objective:**

Breast cancer is the leading cause of death among women in many countries. Numerous factors serve as oncogenes or tumor suppressors in breast cancer. The large family of Tripartite-motif (TRIM) proteins with ~ 80 members has drawn attention for their role in cancer. *TRIM3* and *TRIM16* have shown suppressive activity in different cancers. This study aimed to evaluate the expression of *TRIM3* and *TRIM16* in cancerous and normal breast samples and to investigate their association with different clinical and pathological parameters.

**Results:**

qRT-PCR was utilized to determine the gene expression of *TRIM3* and *TRIM16*. The expression of *TRIM3* and *TRIM16* genes in tumor samples were significantly reduced to 0.45 and 0.29 fold, respectively. *TRIM3* and *TRIM16* genes expression were both positively correlated with the invasion of breast cancer. *TRIM3* gene expression was associated with tumors’ histological grade. However, no significant association was found between the expression of the genes and tumor size, stage and necrosis. The expression of *TRIM3* and *TRIM16* are significantly reduced in breast cancer tissues. Besides, the expression of both *TRIM3* and *TRIM16* genes significantly plummet in lymphatic/vascular and perineural invasive samples. Hence, we suggest a potential tumor suppressor role for *TRIM3* and *TRIM16* in breast cancer.

## Introduction

Breast cancer (BC) is known as one of the most lethal cancers among women [1]. The development of breast cancer and its progression depends on various factors such as genetic and epigenetic factors, lifestyle, and family history. Thus, the incidence and mortality rates of BC vary in different regions and are different among women of different races [2, 3].

In early stages, BC is non-invasive and the tumor cells lack metastatic ability. Over time, if left untreated, the breast tumor grows, and as a result of EMT, the cancer cells become metastatic [4]. According to the TNM staging system, the stages of breast cancer progression are divided into four steps, which a higher stage indicates more tumor volume and more invasive cancer cells [5].

Oncogenes and tumor suppressors such as *c-Myc* and *p53*, respectively, are important in cancer development; hence, they attract much attention in cancer studies [6, 7]. However, some factors play different roles in different types of cancers. Through their ubiquitin E3 ligation activity, Tripartite-motif protein (TRIM) family proteins play a significant role in important cellular processes such as cell development, apoptosis, innate immunity, and autophagy [8]. Therefore, in most cases, dysregulated activity or impaired gene expression of these factors leads to cancer [9]. The association of different members of TRIM proteins with various cancers has been previously shown [10, 11].

*TRIM3* and *TRIM16* are important members of the TRIM family, whose roles have been studied on innate immunity, autophagy, and carcinogenesis [12, 13]. However, there is a clear duality in the role of these factors in different cancers. Huang et al. and Song et al. suggested that *TRIM3* is a tumor suppressor in the liver and cervical cancers, respectively [14, 15]. However, Wang et al. reported that *TRIM3* plays a stimulating role in the MCF7 breast cancer cell line [16]. On the other hand, the study conducted by Marshall et al. suggests that *TRIM16* acts as a tumor suppressor in neuroblastoma cells [17]. However, Yan et al. reported that overexpression of *TRIM16* enhances the metastasis of gastric cancer cells [18].

Regarding the roles of *TRIM3* and *TRIM16* in breast cancer, a limited number of studies have been conducted, most of which are are in vitro. There are mixed results on the role of *TRIM3* in breast cancer, some of which reporting a tumor suppressor role for the enzyme and a few suggesting an oncogenic activity. Yongzhen Li reports in his study that the oncogenic miR-4513 plays its role in MCF-7 cell line through inhibition of *TRIM3*, which leads to increased cell migration, invasion, colony formation and proliferation [19]. However, the study of Wang introduces the *TRIM3* as an oncogenic factor in breast cancer, which promotes the proliferation of breast cancer cells through suppression of P53 signaling [20]. *TRIM16* is suggested by Kim as a suppressor factor in breast cancer that reduces the viability of breast cancer cells [21]. And the study of Yao, working on the effects of *TRIM16* in breast cancer cells and tissue samples, implies that *TRIM16* expression is lowered in breast cancer tissues, and that the enzyme inhibits the proliferation and properties of breast cancer stem cells (CSCs) [22].

Since the previous studies have not made it clear that what roles *TRIM3* and *TRIM16* play in breast tumors, in terms of oncogenic and tumor-suppressor activities, this study aimed to evaluate the expression of *TRIM3* and *TRIM16* genes in breast cancer tissue samples of Iranian women with unique demographic characteristics, prepared in Tehran, Iran, and to compare them to correspondent normal tissues, and also to investigate their roles in breast cancer and their relationship with cancer stage and metastasis.

## Main text

### Materials and methods

#### Tissue specimen collection

40 cancerous breast tissue samples paired with the same number of normal adjacent tissue samples were obtained from the Cancer Institute of Imam Khomeini hospital (Tehran, Iran). The samples were collected from 40 Iranian female patients with breast cancer, most of which were of Persian, Turk, Kurd, Lor, and Gilaki/Mazani races. The average of the patient’s ages was 51.6 ± 10.3 years. By the time of this study, no patient had undergone chemotherapy or radiotherapy. The samples were collected from different sites of the breast including ducts, lobules, nipples, and local lymphatic nodes. For later experiments, each sample was stored in RNAlater, immediately after the tissue was removed. The clinical and pathological information of each sample including histological grade and TNM staging was determined by the pathologist through established protocols.

Each patient has declared her agreement with the sample collection through a consent letter. This study was conducted in accordance with Helsinki declaration and Good Clinical Practices guideline and is approved by the ethics committee of the cancer institute of Imam Khomeini hospital (Tehran, Iran) and the ethics committee of Ahvaz Jundishapour University of medical sciences (Ahvaz, Iran). Complete demographic, clinical and pathological information of patients are presented in Table 1.

**Table 1:** Demographic information of patients

Parameters	Patients group (%)
Age (years)	
< 50	57.5
≥ 50	42.5
Race	
Persian	22.5
Turk	30
Gilaki and Mazani	10
Kurd	12.5
Lor	7.5
N/A	17.5
Histology grade	
Grade I (low-well differentiated)	17.5
Grade II (intermediate-moderately differentiated)	47.5
Grade III (high-poor differentiated)	35
Stage	
II	72.5
III	27.5
Necrosis	
Yes	65
No	35
Vascular/lymphatic invasion	
Positive	62.5
Negative	37.5
Perineural invasion	
Yes	30
No	70

#### Total RNA extraction and cDNA synthesis

Total RNA was extracted from all 80 frozen tissue samples using Hybrid-R RNA isolation kit (GeneAll, Songpa-gu, Seoul, South Korea) according to the manufacturer’s instructions. The product concentration and purity were determined using Nanodrop 2000 instrument (Thermo Fisher Scientific, Wilmington, DE, United States). 1.5% Agarose gel electrophoresis was utilized to evaluate the integrity of isolated RNA. cDNA was synthesized through reverse transcription in 20 μL reaction mix using cDNA synthesis kit (Yekta Tajhiz Azma, Tehran, Iran) according to manufacturer’s instructions. The end products were stored at − 20 °C for further usage.

#### Real-time qRT-PCR

The relative expression of *TRIM3* and *TRIM16* genes were evaluated through qRT-PCR using SYBR green kit (Yekta Tajhiz Azma Tehran, Iran) on ABI Step One Plus instrument (Thermo Fisher Scientific, Waltham, Massachusetts, United States). The *HPRT* (Hypoxanthine–Guanine Phosphoribosyl-transferase) gene was selected as the internal reference. The primer sequences used for PCR reaction are as follows: *HPRT* F: 5′-GACCAGTCAACAGGGGACAT-3′, R: 5′-CCTGACCAAGGAAAGCAAAG-3′, *TRIM3* F: 5′-GCGACCTGGAGACCATTTGT-3′, R: 5′-GCTACTGCCGATGTGTTCCTG-3′, *TRIM16* F: 5′-GGGAAAGAGGTCCTGTGTGA-3′, R: 5′-GTATCGCCAGTTGTGGTCCT-5′. The reaction cycles were set as 95 °C for 15 min, for one cycle, 95 °C for 15 s and 60 °C for 1 min, for 40 cycles. The reaction efficiency for all genes was calculated using LinRegPCR software. Since the efficiency of all genes was > 90%, the Ct numbers were converted to fold change through 2^−∆∆Ct^ for further analysis.

#### Statistical analysis

The statistical analysis of the data was conducted using the IBM SPSS 26.0 software. The normality of the collected data was assessed through Kolmogorov–Smirnov and Shapiro–Wilk tests. The results of the experimental groups were compared using the Kruskal–Wallis and Mann–Whitney U tests. The p-values less than 0.05 were considered significant.

### Results

#### TRIM3 gene expression

**Fig. 1:**
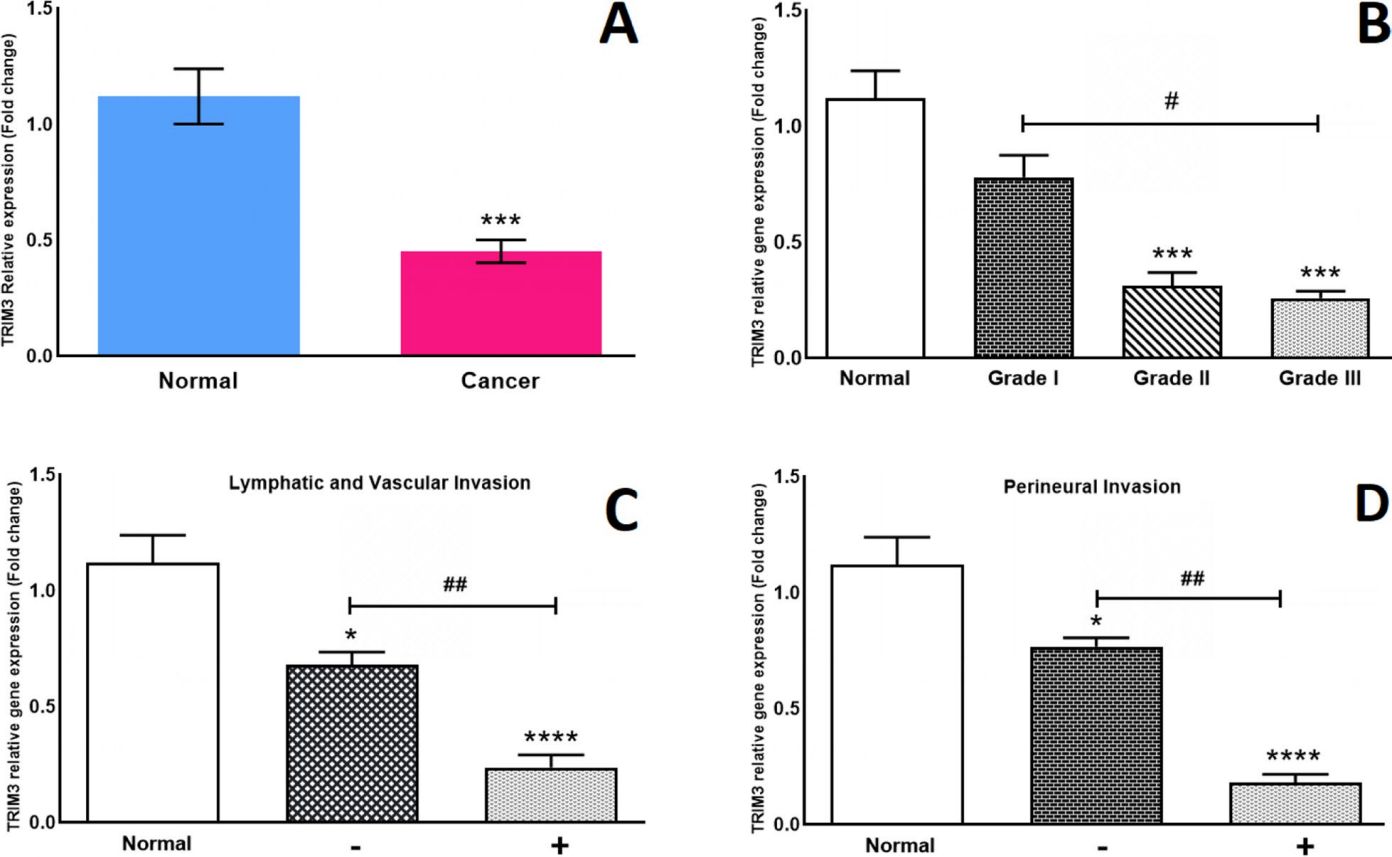
Relative gene expression of *TRIM3* (Tripartite-motif-3) in breast cancer samples compared with normal breast tissues and evaluation of relative gene expression of *TRIM3* in 3 different clinical and pathological parameters through Real-time PCR. **A** The expression of the *TRIM3* gene was significantly decreased in breast cancer tissues. **B** Relative gene expression of *TRIM3* was evaluated in different grades of breast cancer. **C** Relative expression of *TRIM3* was compared between the lymphatic/vascular invasive group and the non-invasive group. **D** Relative expression of *TRIM3* was compared between perineural invasive group and non-invasive group. *p < 0.05, ***p < 0.001, ****p < 0.0001 significantly different from normal group. ^**#**^p < 0.05, ^**##**^< 0.01 significantly different from the selected group

qRT-PCR was utilized to investigate whether the expression levels of *TRIM3* and *TRIM16* were altered in cancerous samples compared to normal tissues. According to our results, the mean fold change of *TRIM3* gene expression in cancer tissues was ~ 0.45, which suggests a ~ 65% reduction in cancer tissues compared to the normal ones (Fig. 1A). Next, the relationship between the expression of the *TRIM3* gene and the clinical and pathological status of cancer tissues was evaluated. Our results showed that although the expression of *TRIM3* was not significantly reduced in grade I samples, the results of grade II and III showed that it is reduced to 0.31 and 0.26 fold, respectively (Fig. 1B). Also, the expression of *TRIM3* was compared between the samples with and without lymphatic/vascular invasion (LVI). *TRIM3* gene expression was decreased to 0.68 and 0.24 fold in LVI− and LVI+ samples, respectively (Fig. 1C). Another factor with which the expression of *TRIM3* was compared, was perineural invasion (PI). Our results showed that the *TRIM3* gene expression in PI− and PI+ groups was 0.76 and 0.18 fold, respectively (Fig. 1D). Further clinical and pathological factors including TNM stage, tumor size, and necrosis were used to evaluate *TRIM3* gene expression, none of which showed a significant difference between different states of each experimental group (Table 2).

**Table 2:** Association of *TRIM3* and *TRIM16* genes expression with breast cancer clinical and pathological characteristics

Variables	*TRIM3*		*TRIM16*	
	Mean fold change	~ p-value	Mean fold change	~ p-value
Tumor size (cm)				
< 5	0.52	0.999	0.29	0.999
≥ 5	0.38		0.31	
Grade				
I	0.77	0.029*	0.27	0.192
II	0.31		0.36	
III	0.25		0.22	
Stage				
II	0.58	0.240	0.33	0.145
III	0.34		0.26	
Necrosis				
Yes	0.48	0.508	0.21	0.281
No	0.41		0.36	
Lymphatic/vascular invasion				
Yes	0.67	0.006**	0.16	0.044*
No	0.28		0.42	
Perineural invasion				
Yes	0.60	0.003**	0.13	0.025*
No	0.25		0.47	

*p < 0.05, **p < 0.01 significantly different from the opposite group

#### TRIM16 gene expression

**Fig. 2:**
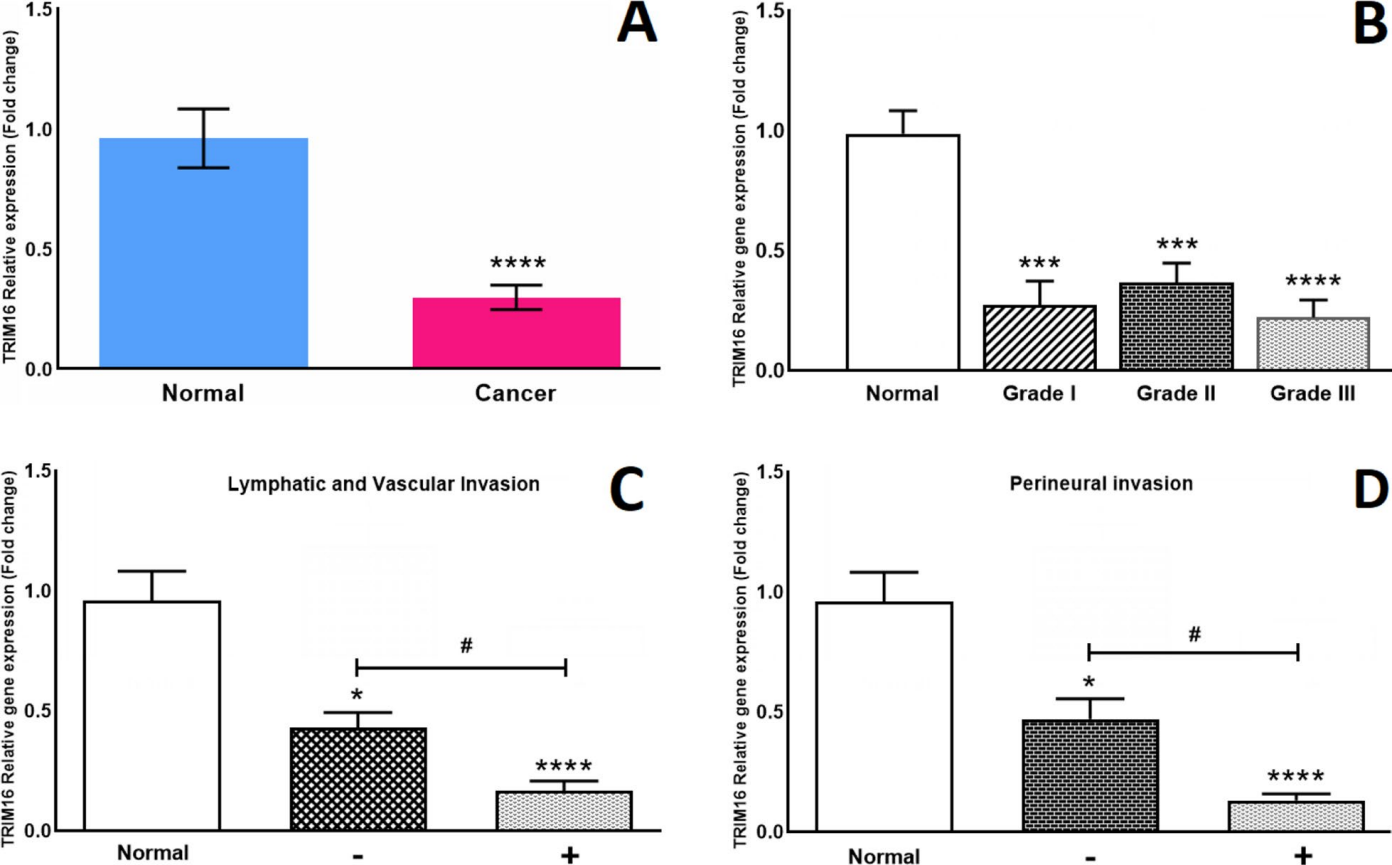
Relative gene expression of *TRIM16* (Tripartite-motif-16) in breast cancer samples in comparison with normal breast tissues and evaluation of relative gene expression of *TRIM16* in 2 different clinical and pathological parameters through Real-time PCR. **A** The expression of the *TRIM16* gene was significantly decreased in breast cancer tissues. **B** Relative gene expression of *TRIM3* was evaluated in different grades of breast cancer. **C** Relative expression of *TRIM16* gene was compared between lymphatic/vascular invasive group and non-invasive group. **D** Relative expression of *TRIM16* was compared between perineural invasive and non-invasive group. *p < 0.05, ***p < 0.001, ****p < 0.0001 significantly different from normal group. ^**#**^p < 0.05 significantly different from the selected group

Firstly, the expression of the *TRIM16* gene in the cancer group was compared with that of the normal group. According to our results, presented in Fig. 2A, the expression of the *TRIM16* gene in the cancer group was reduced to 0.29 fold, which shows a significant ~ 67% drop. *TRIM16* gene expression showed no significant difference between different grades of breast cancer (Fig. 2B). Furthermore, our results showed that the *TRIM16* gene expression in LVI− and LVI+ groups were 0.42 and 0.16 fold respectively (Fig. 2C). Lastly, the expression of TRIM16 was compared between PI− and PI+ groups, which the latter was decreased to 0.13 fold (Fig. 2D). The expression of *TRIM16* showed no significant difference between different states of each experimental group in terms of tumor size, cancer grade, TNM stage, and necrosis (Table 2).

### Discussion

The large protein family of TRIMs have been widely studied in terms of cancer. TRIM proteins are largely involved in important cellular processes such as cell growth and differentiation [9, 23]. Furthermore, the TRIM family members may leave their mark in carcinogenesis through their association with important cancer-related factors, such as p53 and TGF-β [24, 25].

*TRIM3* and *TRIM16* are two important members of the TRIM family, both of which have shown inhibitory effects in different types of cancer, however a few studies have reported oncogenic activities for them. A study by Hailong et al. reported that the knocked down *TRIM3* leads to promoted growth and metastasis of gastric cancer cells, and that *TRIM3* can play the role of a biomarker for gastric cancer diagnosis [26]. According to a report by Mei-yu et al., *TRIM3* serves as a tumor suppressor in colorectal cancer and it may be a potential therapeutic marker for CRC [27]. Also, Nagy et al. reported that *TRIM16* expression is down-regulated through the transition of normal skin cells to squamous cell carcinoma, which suggests a tumor suppressive role for the enzyme [28]. There are several diverse mechanisms through which *TRIM3* and *TRIM16* act against carcinogenesis. *TRIM16* is reported to be involved in cellular anti-oxidant mechanisms through Nrf2/ARE signaling, which may play a major role against cancer [29]. Furthermore, the up-regulation of the *TRIM16* gene leads to the down-regulation of several genes, such as *MMP*-*2*, *MMP*-*9*, *Smo*, and *Gli*-*1*, which are highly involved in cancer cell invasion [30]. Similarly, *TRIM3* is reported to inactivate the highly cancer-related p38 pathway, thus, enacting its role opposite cancer [15].

In this study, we investigated the expression of *TRIM3* and *TRIM16* genes in normal and breast cancer tissue samples and compared the expression of the two genes between different clinical and pathological states. According to our results, the expression of *TRIM3* and *TRIM16* genes in the cancer group undergoes a significant reduction to 0.45 and 0.29 fold, respectively. These results are in line with the previous studies suggesting that *TRIM3* and *TRIM16* were down-regulated in different types of cancer, which supports the idea of the tumor suppressor role for the enzymes [31, 32].

Next, we used six different clinical and pathological parameters, including tumor size, necrosis, histological grade, TNM stage, lymphatic/vascular invasion, and perineural invasion to gain a better view of the effects of *TRIM3* and *TRIM16* through the progression breast cancer. Our results showed that the expression of *TRIM3* in grade I tissues was 0.76 fold, which statistically is not a significant reduction. However, in grades II and III, the *TRIM3* gene expression was significantly reduced to 0.31 and 0.26 fold, respectively. These results are in line with the previous work reporting association between *TRIM3* expression and cancer grade[33]. The result of the comparison of *TRIM3* gene expression between LVI+ and LVI− showed a significant reduction of 0.45 fold in LVI+ compared to LVI− group. Besides, the expression of *TRIM3* showed a significant 0.58 fold drop in PI+ compared to the PI− group. These results agree with the studies suggesting inhibitory effects for *TRIM3* on the invasive potential of cancer cells [27]. The *TRIM3* gene expression showed no significant difference between groups in terms of tumor size, necrosis, and TNM stage. However, since all tumor samples obtained were either stage II or III, the levels of *TRIM3* gene expression in stage I and IV remain unclear. On the other hand, the *TRIM16* gene expression showed a significant drop of 0.26 fold in LVI+ compared to LVI− group. Besides, in the PI− and PI+ groups, the expression of the *TRIM16* gene was 0.47 and 0.13 fold, respectively, which demonstrates a significant difference of 0.34 fold between the two groups. These results suggest that *TRIM16* may play important roles in the inhibition of cancer cell metastasis, hence, agree with the previous work [34].

### Conclusion

In this study, we found that *TRIM3* and *TRIM16* are both down-regulated in breast cancer, and in addition, our results demonstrated that *TRIM3* is highly associated with breast cancer grade. Also, we found that both *TRIM3* and *TRIM16* undergo more remission in invasive breast tissues, which may suggest an anti-metastatic role for the two genes. Eventually, we propose *TRIM3* and *TRIM16* as potential tumor suppressors in terms of breast cancer. However, more studies are required to determine the specific roles of the enzymes.

### Limitations

Here, we explored the association between *TRIM3* and *TRIM16* gene expression and the factors that show the progression of BC. However, due to financial limitations we were not able to evaluate protein levels of the factors. Although, we cannot explain the molecular pathways associated with the genes’ function, we consider these genes as “potential” tumor suppressors in BC.

## Data Availability

Not applicable.

## Abbreviations

BC: Breast cancer
TRIM: Tripartite-motif
LVI: Lymphatic-vascular invasion
PI: Perineural invasion
MMP: Matrix-metalloproteinase
CRC: Colorectal cancer
